# Artificial intelligence-based morphologic classification and molecular characterization of neuroblastic tumors from digital histopathology

**DOI:** 10.21203/rs.3.rs-4396782/v1

**Published:** 2024-06-04

**Authors:** Mark Applebaum, Siddhi Ramesh, Emma Dyer, Monica Pomaville, Kristina Doytcheva, James Dolezal, Sara Kochanny, Rachel Terhaar, Casey Mehrhoff, Kritika Patel, Jacob Brewer, Benjamin Kusswurm, Arlene Naranjo, Hiroyuki Shimada, Elizabeth Sokol, Susan Cohn, Rani George, Alexander Pearson

**Affiliations:** University of Chicago; University of Chicago; University of Chicago Medical Center; Children's Hospital of Phildelphia; University of Chicago; University of Chicago Medicine; University of Chicago; University of Chicago; University of Utah; University of Washington; University of Chicago; University of Chicago; University of Florida; Stanford University; Ann & Robert H. Lurie Children's Hospital of Chicago; University of Chicago; Harvard Medical School; University of Chicago

## Abstract

A deep learning model using attention-based multiple instance learning (aMIL) and self-supervised learning (SSL) was developed to perform pathologic classification of neuroblastic tumors and assess *MYCN*-amplification status using H&E-stained whole slide digital images. The model demonstrated strong performance in identifying diagnostic category, grade, mitosis-karyorrhexis index (MKI), and *MYCN*-amplification on an external test dataset. This AI-based approach establishes a valuable tool for automating diagnosis and precise classification of neuroblastoma tumors.

## Introduction

Neuroblastoma is a neuroblastic tumor (NT) and the most common extracranial pediatric solid tumor, affecting nearly 800 children in the United States annually.^1^ To select optimal treatment strategies, patients are risk-stratified according to prognostic clinical, pathologic, and molecular variables including age, stage, histopathology, and *MYCN*-amplification.^2,3^ Approximately 40% of patients with neuroblastoma are classified as high-risk, which carries a 60% overall three-year likelihood of event free survival.^4^ *MYCN*-amplification is present in 20% of NTs and, when identified, places the patient in the high-risk category.^5^

The pathologic classification of NTs is a major contributor to risk stratification. The International Neuroblastoma Pathology Committee (INPC) uses combinations of four features–age, diagnostic category (neuroblastoma, ganglioneuroblastoma intermixed, ganglioneuroma, or ganglioneuroblastoma nodular), grade of differentiation, and mitosis-karyorrhexis index (MKI) – to classify tumors as favorable or unfavorable histology.^6^ INPC classification has significant prognostic ability unto itself, as those with unfavorable histology have a four times higher likelihood of relapse compared to those with favorable histology.^2^

Histology from hematoxylin and eosin (H&E)-stained slides can also serve as a rich data source for deep learning models, which can be used to identify nuanced motifs in tumor morphology and produce precise risk stratification criteria.^7-9^ Machine learning algorithms have been used to analyze NT digitized histology as early as 2009, with models that segmented cells and extracted texture features from histology images to predict tumor grade.^10^ More recently, convolutional neural networks (CNNs) have been deployed on NT histology risk stratification.^11^

**Figure 1:**
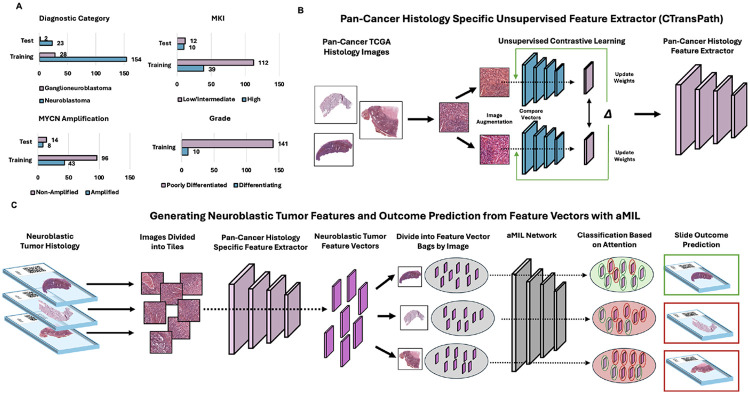
attention-based multiple instance learning (aMIL) models use feature vectors as inputs, grouped in bags, to make predictions aggregated from all vectors within a bag. (**A**) Number of slides in the training and test cohorts by pathologic category. (**B**) Models were pre-trained with histology-specific digital images using unsupervised domain-specific learning to extract features with CTransPath. (**C**) Whole slide images (WSI) were divided into tiles, passed through the fine-tuned network to generate neuroblastoma-specific feature vectors, which are divided into bags per WSI. The aMIL network assigns attention scores to vectors, and a slide-level prediction is determined based on the aggregated predictions weighted by attention scores.

Using our open-source deep learning analysis pipeline, Slideflow (2.3.1), we developed an attention-based multiple instance learning (aMIL) model with features extracted by CTransPath, a pre-trained self-supervised learning (SSL) model.^12-14^ In contrast to conventional CNNs, aMIL models rely on pre-trained features to begin model training (Fig. 1). These features are obtained by passing images through a feature extractor network that has been pre-trained on either domain-specific or non-specific images. CTransPath is a domain-specific model that has been trained on unlabeled H&E-stained slides from The Cancer Genome Atlas (TCGA).^12^ For limited datasets such as those obtainable in rare diseases, using domain-specific features to train an aMIL can offer significant performance advantages over non-specific models such as ImageNet.^15,16^

In this study, we leveraged the largest reported study cohort of digitized NTs analyzed with these state-of-the-art deep learning methods. We generated a training dataset of whole slide images (WSIs) from patients from the University of Chicago and the Children’s Oncology Group. These WSIs were used to develop models for predicting diagnostic category, grade, MKI, and *MYCN*-amplification status. Model performance was validated on an external test dataset of WSIs from patients seen at Lurie Children’s Hospital. We aimed to demonstrate the feasibility of using aMILs to aid in NT classification and risk stratification.

The median age of patients with digitalized NT in the training dataset (n = 172) was 2.63 years (SD = 4.37). Among patients with additional known clinical information, 84 of 138 (60.2%) had metastatic disease and 94 of 133 (70.7%) were high-risk. For diagnostic category, the dataset includes 24 ganglioneuroblastomas and 148 neuroblastomas which were confirmed by pathologists (KD, HS, PP). Of the 148 tumors with a diagnostic category of neuroblastoma, 93.2% were poorly differentiated and 25% had high MKI. Of the 135 tumors with known *MYCN* status, 40 were amplified (29.6%). The median age of the external test dataset (n = 25) was 3.33 years (SD = 2.90). All patients in the test dataset were high-risk and 23 of 25 (92%) had metastatic disease. Of the 23 tumors classified as neuroblastoma, all were poorly differentiated. Eleven of these 23 tumors (48%) had a high MKI. Eight of the 25 tumors (32%) were *MYCN*-amplified.

**Figure 2:**
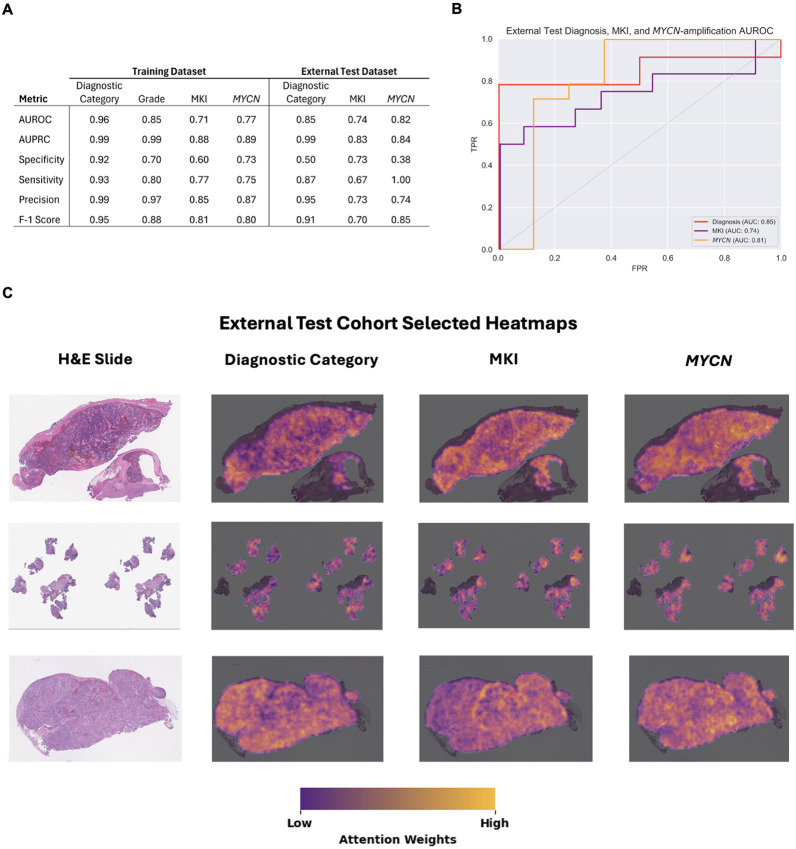
Model performance and explainability. (**A**) Performance metrics for training and external test models. (**B**) AUROC plots for the external test models. (**C**) Explainability heatmaps generated with GradCAM. Yellow regions were highly weighted and informative to the model while dark purple regions corresponded to low weights in generating predictions. Abbreviations: AUROC, Area Under the Receiver Operator Curve; AUPRC, Area Under the Precision Recall Curve.

The final models demonstrated highly accurate performance across all outcomes in the training cohort (Fig. 2). Area Under the Receiver Operator Curve (AUROC) for diagnostic category, grade, MKI, and *MYCN* were 0.96, 0.85, 0.71, and 0.77, respectively, and (Area Under the Precision Recall Curve) AUPRC was 0.99, 0.99, 0.88, and 0.89, respectively. The model had the most success identifying diagnostic categories, with a sensitivity of 0.93 and specificity of 0.92. For *MYCN* status, a sensitivity of 0.75 and specificity of 0.73 was demonstrated in the analysis.

Using an independent cohort of clinically annotated NT tumors, the models demonstrated high accuracy across all analyzed outcomes, validating the findings in the training data set (Fig. 2). For diagnostic category, the AUROC was 0.85 [95% Confidence Interval (CI) 0.71–0.99], with an AUPRC of 0.99 (95% CI 0.94–1.0), sensitivity of 0.87 (95% CI 0.68–0.95), and specificity of 0.50 (95% CI 0.09–0.91). The AUROC for MKI was 0.74 (95% CI 0.56–0.92), with an AUPRC of 0.83 (95% CI 0.68–0.99), sensitivity of 0.50 (0.25–0.75), and specificity of 0.91 (95% CI 0.62–0.98). For MYCN status, the AUROC was 0.81 (95% CI 0.65–0.98), with an AUPRC of 0.77 (95% CI 0.64–0.97), sensitivity of 1.0 (95% CI 0.78-1.0), and specificity of 0.63 (95% CI 0.30–0.86). Grade could not be assessed in the external test cohort as all samples were poorly differentiating.

Expert pathologist (PP) review of the model's attention heatmaps, generated using GRAD-CAM, revealed that the models were primarily focusing on neoplastic areas of the tumor, rather than relying on non-tumor tissues such as fibrosis, fibrovascular stroma, or adrenal tissue. While in most cases the model accurately identified and focused on the relevant tumor regions, in some instances correlation was unevenly distributed across the relevant tumor area. This suggests that this variation in attention may correlate with less well characterized diffuse histopathological signatures that have unclear associations with standard pathologic descriptions. Further investigation into these attention patterns is necessary to elucidate novel morphological features or subtypes within neuroblastoma tumors.^17^ Overall, the pathologist's analysis confirmed that the model was generally making predictions based on the most relevant areas within the neoplastic regions of each sample.

We show the feasibility of using small datasets of H&E-stained WSIs to develop models for morphologic classification of NTs and accurate assessment of *MYCN*-amplification status at diagnosis using an aMIL deep learning model. While prior deep learning models for NTs relied heavily on morphological feature extraction and labeled data, our method used unlabeled data in conjunction with SSL methods to improve model performance when working with a small dataset.^10,11^ The model achieved notable performance in identifying diagnostic category and a strong ability to identify *MYCN*-amplification. The highly accurate automatic classification produced by the model could be refined with additional data to eventually streamline pathologist workflows.

The model’s ability to identify *MYCN*-amplification status from histology is an encouraging result, particularly given the limited data used to train the model. This suggests models could also be built to predict other relevant genomic features such as copy number variations and ploidy. As 50% of high-risk NTs do not harbor *MYCN*-amplification and typically have other findings such as 11q aberrations, a deep learning approach may also provide the ability to readily identify features that drive aggressive growth in non-*MYCN*-amplified high-risk tumors.^18^ Unlike immunohistochemistry or fluorescence *in situ* hybridization where a single gene aberration is probed, deep learning models analyze the image at a global level and may be able to more readily identify morphological signatures produced by combinations of gene alterations that could further aid in stratifying NTs.

Limitations of this study arise largely from data availability. As NTs are rare, it remains difficult to collect sufficient samples to train a robust deep learning model. Our approach makes use of a network architecture that seeks to overcome this limitation. However, the model could further be improved with more data. Additionally, this study seeks to aid molecular pathology diagnostics and does not constitute a pathologist replacement. The model’s predictions act as a second pair of eyes and could alert a pathologist to review specific, notable aspects of the histology.

This work provides an important step forward in automating diagnosis and precise classification of NTs with the addition of deep learning-based image analysis. Ultimately, this can increase global access to molecular and pathological classification for tumors in regions without access to experts. We also demonstrate the ability of aMIL models to perform well on small datasets; this model architecture could be extended to other rare cancers that suffer from low data availability. This artificial intelligence-based approach establishes another data modality in the pathologist’s toolbox for NT classification.

## Methods

### Dataset description

H&E-stained slides from the time of initial diagnosis were obtained from the University of Chicago (n = 102), the Children’s Oncology Group (n = 70), and Lurie Children’s Hospital (n = 25). The images were reviewed by trained pathologists (HS, PP, KD) who annotated the tumor regions and defined the diagnostic category (ganglioneuroblastoma/neuroblastoma), grade (differentiating/poorly differentiating), and MKI (low/intermediate and high). *MYCN* status was abstracted from patient records (amplified/non-amplified). This study was approved by the University of Chicago (IRB20-0659) and Lurie Children’s Hospital Internal Review Boards (IRB 2021–4498).

### Image processing

WSIs were captured using an Aperio AT2 DX WSI Scanner. To remove normal background tissue and maximize cancer-specific training, image tiles were extracted from within pathologist-annotated regions of tumor. Image tiles were extracted from WSIs with a width of 302μ and 299 x 299 pixels using Slideflow version 2.3.1 and the libvips backend. Grayspace filtering, Otsu’s thresholding, and gaussian blur filtering (sigma = 3, threshold = 0.02) were used to remove background.

### Classifier training

Extracted tiles were converted into feature vectors using CTransPath with ‘reinhard mask’ normalization applied.^12^ aMIL models were trained on extracted features in Slideflow with the FastAI API and Pytorch. The aMIL model parameters were: weight decay of 1e^− 5^, bag size of 256, batch size of 32, and training for 10 epochs. aMIL models were evaluated with 5-fold cross validation and by calculating the average AUROC, AUPRC, sensitivity, specificity, and F-1 score. Patients were excluded from a given model if the measure of interest was unknown.

### Model Validation

The aMIL model developed during training was used on the unseen external test dataset. Samples were evaluated in one run without any hyperparameter tuning on test data to ensure no validation leakage. Model performance was assessed as above.

### Pathologist Explainability Assessment

Explainability heatmaps were generated using GradCAM.^19^ PP reviewed the heatmaps to identify whether tumor regions that the model found important for outcome prediction had clinical correlation to the given outcome.
